# Assessing the content validity of the revised Health of the Nation Outcome Scales 65+: the HoNOS Older Adults

**DOI:** 10.1192/bjb.2022.37

**Published:** 2023-08

**Authors:** Meredith G. Harris, Caley Tapp, Urska Arnautovska, Tim Coombs, Rosemary Dickson, Mark Smith, Angela Jury, Jennifer Lai, Mick James, Jon Painter, Philip M. Burgess

**Affiliations:** 1 The University of Queensland, Brisbane, Australia; 2 Australian Mental Health Outcomes and Classification Network, Brisbane, Australia; 3 Australian Mental Health Outcomes and Classification Network, Sydney, Australia; 4 Te Pou, Hamilton, New Zealand; 5 Te Pou, Auckland, New Zealand; 6 Royal College of Psychiatrists, London, UK; 7 Sheffield Hallam University, Sheffield, UK

**Keywords:** Older adults, routine outcome measurement, content validity, measurement properties, mental health services

## Abstract

**Aims and method:**

Recently, the Health of the Nation Outcome Scales 65+ (HoNOS65+) were revised. Twenty-five experts from Australia and New Zealand completed an anonymous web-based survey about the content validity of the revised measure, the HoNOS Older Adults (HoNOS OA).

**Results:**

All 12 HoNOS OA scales were rated by most (≥75%) experts as ‘important’ or ‘very important’ for determining overall clinical severity among older adults. Ratings of sensitivity to change, comprehensibility and comprehensiveness were more variable, but mostly positive. Experts’ comments provided possible explanations. For example, some experts suggested modifying or expanding the glossary examples for some scales (e.g. those measuring problems with relationships and problems with activities of daily living) to be more older adult-specific.

**Clinical implications:**

Experts agreed that the HoNOS OA measures important constructs. Training may need to orient experienced raters to the rationale for some revisions. Further psychometric testing of the HoNOS OA is recommended.

The clinician-rated Health of the Nation Outcome Scales 65+ (HoNOS65+) was first published in 1999.^1,2^ It was adapted from the HoNOS for working-age adults^3^ based on feedback that specific content changes were needed to meet the needs of older adults.^4,5^ The HoNOS65+ comprises 12 scales that cover the types of problem experienced by older adults in contact with specialised mental health services, equivalent to the scales in the working-age version.^3^ Maximum severity is rated (usually) for the previous 2 weeks, with ratings guided by a glossary.

Following a review of the HoNOS for working-age adults,^6^ the Royal College of Psychiatrists invited individuals with expertise in working with older adults to join an advisory board to propose amendments to the HoNOS65+. The advisory board comprised representatives from England, Australia and New Zealand with extensive experience in using the HoNOS in staff training, clinical practice, service monitoring and governance. The board drew on their own experience, as well as the views of clinicians in their professional networks, to identify aspects of the measure requiring refinement. Proposed amendments were judged against criteria developed by the board, one of which was to retain as far as possible the structure and core rules of the original measure.^7^ Both the HoNOS and HoNOS65+ were revised with the intent of reducing ambiguity and inconsistency in the glossaries and improving reliability, validity and utility. For scales where it was considered that presenting needs were the same regardless of age, the wording of the two glossaries was made more consistent. The revised HoNOS65+ was named the HoNOS Older Adults (HoNOS OA),^7^ reflecting a shift towards later onset of functional impairment^8^ and because age cut-offs for older adult services can vary between services and over time.

The HoNOS OA was published in 2018 and, as yet, there is no empirical evidence about its measurement properties. When a measure is revised, the assessment of content validity – whether the content of a measure adequately reflects the construct(s) of interest – is recommended as the first step because deficits in content validity may affect other properties.^9^ For multidimensional measures the content validity aspects of relevance, comprehensiveness and comprehensibility should be assessed for each item.^9^ We designed and conducted a study of the content validity of the 12 HoNOS OA scales.

## Method

This descriptive study involved completion of an anonymous web-based survey by experts from Australia and New Zealand. Experts were identified through database bibliographic searches and professional networks. None of the experts invited to complete the survey were members of the advisory board that proposed amendments to the HoNOS65+ and produced the revised set of scales known as the HoNOS OA. Expertise was defined as: making or supervising HoNOS65+ ratings; psychometric or clinical effectiveness research involving the HoNOS65+; and/or using HoNOS65+ ratings at a macro level (e.g. staff training, monitoring service quality).

Experts were invited to participate via an email containing a link to the survey (one expert subsequently requested a paper-and-pencil version). The survey began with an information sheet; written informed consent was obtained from all participants. Consenting participants were asked questions about relevant professional characteristics. They were then presented with each scale of the HoNOS OA and asked for their opinion in response to six ‘core’ questions:

1. How important is this scale for determining overall clinical severity for older adult mental health service consumers? (relevance)
2. How likely are repeat ratings on this scale to capture change in [scale-specific problems] during a period of mental healthcare? (relevance)
3. How well do the descriptors for each rating of 0–4 cover the range of [scale-specific problems] typically seen among older adult mental health service consumers? (comprehensiveness)
4. How helpful is the glossary for determining what to include when rating [scale-specific problems]? (comprehensibility)
5. How well do the descriptors for each rating of 0–4 correspond to the different levels of severity of [scale-specific problems]? (comprehensibility)
6. How consistent is the wording of the glossary with language used in contemporary mental health practice? (comprehensibility).

Responses were made on a 4-point Likert scale^10^ (1 Not important; 2 Somewhat important; 3 Important; 4 Very important). Open-ended questions encouraged experts to elaborate on their ‘negative’ ratings (i.e. ratings of 1 or 2). At the end of the survey, experts were invited to make additional comments about the content of the HoNOS OA.

An item-level content validity index (I-CVI)^11,12^ shows the proportion of experts who rated each scale positively on each core question. The I-CVI is calculated by dividing the total number of ‘positive’ ratings (i.e. ratings of 3 or 4) by the number of raters. At the 5% significance level, an I-CVI value ≥0.75 indicates ‘excellent’ content validity when there are ≥16 raters.^11^ The average deviation (AD) index was used to measure the dispersion of responses around the median, with lower values indicating less dispersion.^13^ At the 5% significance level with a 4-point response scale, AD index values ≤0.68 indicate ‘acceptable and statistically significant agreement’ when there are ≥15 raters.^13^ Statistical analyses were conducted in Stata 16.0 for Windows (StataCorp, College Station, TX, USA). Open-ended comments were analysed independently by two members of the research team using template analysis.^14,15^ The initial coding template was based on themes arising from a concurrent study of the content validity of the revised HoNOS for working age adults (HoNOS 2018),^16^ then refined iteratively as the comments were coded. The final template was applied across all comments.

Each site received approval to conduct the study and to pool the data for analysis: Australia (University of Queensland Medicine, Low & Negligible Risk Ethics Sub-Committee, 2019/HE002824; Research Ethics and Integrity, 2021/HE000113); New Zealand (ethics review not required; Ministry of Health, Health and Disability Ethics Committees).

## Results

Of 35 invited experts, 25 completed the survey (71% response rate). Most (72%) were psychiatrists or nurses; the remainder represented a mix of disciplines. Experts represented the three types of expertise sought and, collectively, had used the HoNOS65+ across a mix of settings. A quarter said they had used the HoNOS OA in their work (Table 1).

**Table 1:** Characteristics of experts who completed the survey (*n* = 25)

	*n*	%
Main professional background		
Psychiatrist	11	44
Nurse	7	28
Clinical psychologist	3	12
Other^a^	4	16
Expertise in working with HoNOS65+^b^		
Rating HoNOS65+ or reviewing HoNOS65+ ratings made by others	23	92
Research in the measurement properties of HoNOS65+ and/or measuring clinical effectiveness	3	12
HoNOS65+ staff training and/or using HoNOS65+ results at a macro level	15	60
Other expertise working with HoNOS65+	4	16
Mental health settings worked with HoNOS65+^b^		
In-patient	17	68
Community services	23	92
Other setting^c^	4	16
Aware of HoNOS OA prior to survey		
No, I was not aware of the HoNOS OA at all	12	48
Yes, I was aware of the HoNOS OA, but have not used it in my work	6	24
Yes, I have used the HoNOS OA in my work	6	24
Not sure	1	4
	Mean (s.d.)	Range
Years worked in mental health	24.1 (10.8)	2–42
Years worked with the HoNOS65+	13.9 (7.1)	2–28

HoNOS OA, Health of the Nation Outcome Scales Older Adults.

a. Professions included occupational therapist, social worker, consumer/carer/family advisor/leader.

b. Categories not mutually exclusive.

c. Other settings included residential (Australian respondents), non-clinical setting.

### Experts’ ratings of relevance, comprehensiveness and comprehensibility

The I-CVI values show that ‘positive’ ratings were made by at least half (i.e. I-CVI ≥ 0.5) of experts on all but one of the core questions and by three-quarters of experts (i.e. I-CVI ≥ 0.75) on nearly 70% of core questions (Tables 2 and 3).

**Table 2:** Experts’ ratings of the content validity of the HoNOS OA scales: relevance and comprehensiveness

HoNOS OA scale	Relevance								Comprehensiveness			
	How important is this scale for determining overall clinical severity for older adult mental health service consumers?				How likely are repeat ratings on this scale to capture change in [scale-specific problems] during a period of mental healthcare?				How well do the descriptors for each rating of 0–4 cover the range of [scale-specific problems] typically seen among older adult mental health service consumers?^a^			
	*n*	Range	I-CVI	AD index	*n*	Range	I-CVI	AD index	*n*	Range	I-CVI	AD index
Scale 1, Overactive or aggressive or disruptive or agitated behaviour	25	1–4	**0.80**	0.76	25	1–4	0.64	0.68	24	2–4	**0**.**75**	0.50
Scale 2, Non-accidental self-injury	23	2–4	**0**.**87**	0.57	24	1–4	0.67	0.58	23	1–4	0.48	0.83
Scale 3, Problem drinking or drug-taking	23	1–4	**0**.**83**	0.52	24	1–4	0.67	0.63	23	1–4	0.57	0.65
Scale 4, Cognitive problems	25	2–4	**0**.**88**	0.60	24	1–4	**0**.**75**	0.50	25	2–4	**0**.**84**	0.40
Scale 5, Physical illness or disability problems	25	2–4	**0**.**88**	0.60	25	1–4	**0**.**76**	0.60	25	1–4	**0**.**76**	0.56
Scale 6, Problems associated with hallucinations and/or delusions	24	2–4	**0**.**92**	0.58	23	1–4	**0**.**87**	0.52	24	2–4	**0**.**88**	0.38
Scale 7, Problems with depressed mood	25	2–4	**0**.**96**	0.44	25	1–4	**0**.**84**	0.48	25	1–4	0.72	0.60
Scale 8, Other mental and behavioural problems	24	1–4	**0**.**92**	0.50	25	1–4	0.72	0.68	25	1–4	**0**.**76**	0.48
Scale 9, Problems with relationships	25	2–4	**0**.**84**	0.64	25	2–4	0.68	0.60	25	1–4	0.68	0.64
Scale 10, Problems with activities of daily living	24	2–4	**0**.**96**	0.50	24	1–4	**0**.**76**	0.52	24	2–4	0.71	0.42
Scale 11, Problems with housing and living conditions	25	1–4	**0**.**80**	0.60	25	1–4	**0**.**88**	0.46	25	1–4	**0**.**76**	0.44
Scale 12, Problems with occupation and activities	25	1–4	**0**.**80**	0.68	24	1–4	**0**.**76**	0.52	25	1–4	0.68	0.60

HoNOS OA, Health of the Nation Outcome Scales Older Adults; I-CVI, item-level content validity index; AD, average deviation. Bold denotes excellent content validity (i.e. I-CVI ≥ 0.75).

a. To fit the wording of Scale 8, the equivalent question for Scale 8 was ‘How well do problems A–O cover the range of other mental and behavioural problems typically seen among older adult mental health service consumers?’

**Table 3:** Experts’ ratings of the content validity of the HoNOS OA scales: comprehensibility

	Comprehensibility											
	How helpful is the glossary for determining what to include when rating [scale-specific problems]?^a^^,^^b^				How well do the descriptors for each rating of 0–4 correspond to the different levels of severity of [scale-specific problems]?				How consistent is the wording of the glossary with language used in contemporary mental health practice?			
HoNOS OA scale	*n*	Range	I-CVI	AD index	*n*	Range	I-CVI	AD index	*n*	Range	I-CVI	AD index
Scale 1, Overactive or aggressive or disruptive or agitated behaviour	25	2–4	**0.84**	0.36	25	2–4	0.60	0.56	23	1–4	0.70	0.43
Scale 2, Non-accidental self-injury	25	1–4	0.72	0.64	24	1–4	0.54	0.63	24	1–4	0.71	0.54
Scale 3, Problem drinking or drug-taking	24	2–4	**0**.**75**	0.46	23	1–4	0.70	0.70	24	2–4	**0**.**79**	0.33
Scale 4, Cognitive problems	25	1–4	**0**.**88**	0.36	24	1-4	**0**.**75**	0.50	25	1-4	0.68	0.52
Scale 5, Physical illness or disability problems	25	2–4	**0**.**84**	0.48	25	1–4	**0**.**80**	0.64	25	1–4	**0**.**84**	0.44
Scale 6, Problems associated with hallucinations and/or delusions	24	1–4	**0**.**79**	0.46	24	2–4	**0**.**79**	0.50	24	2–4	**0**.**96**	0.29
Scale 7, Problems with depressed mood	25	2–4	**0**.**76**	0.60	24	2–4	**0**.**79**	0.50	25	2–4	**0**.**88**	0.36
Scale 8, Other mental and behavioural problems	25	1–4	0.72	0.56	25	1–4	0.68	0.64	24	2–4	**0**.**75**	0.46
Scale 9, Problems with relationships	25	2–4	**0**.**76**	0.48	25	2–4	0.68	0.64	25	1–4	**0**.**80**	0.52
Scale 10, Problems with activities of daily living	25	2–4	**0**.**76**	0.44	24	1–4	**0**.**79**	0.54	25	2–4	**0**.**84**	0.36
Scale 11, Problems with housing and living conditions	25	1–4	**0**.**80**	0.48	25	1–4	**0**.**80**	0.48	24	1–4	**0**.**88**	0.29
Scale 12, Problems with occupation and activities	25	1–4	0.64	0.60	24	1–4	**0**.**75**	0.50	25	1–4	**0**.**76**	0.44

HoNOS OA, Health of the Nation Outcome Scales Older Adults; I-CVI, item-level content validity index; AD, average deviation. Bold denotes excellent content validity (i.e. I-CVI ≥ 0.75).

a. Question text differed across scales; depending on the glossary, ‘what to rate and include’ or ‘what to rate and consider’ was substituted for the phrase ‘what to include’.

b. To fit the wording of Scale 8, the equivalent question for Scale 8 was ‘How helpful is the glossary for determining which other mental and behavioural problem to rate on this scale?’

However, some aspects of content validity were more frequently endorsed than others. For example, all 12 scales met the *a priori* criterion for excellent content validity (I-CVI ≥ 0.75) for the question assessing importance for determining overall clinical severity (Tables 2 and 3). Between six and nine scales met the criterion for all other questions.

Conversely, some HoNOS OA scales met the criterion for excellent content validity more often than others. For example, three scales met the criterion for all questions: Scale 5 (Physical illness or disability problems), Scale 6 (Problems associated with hallucinations and/or delusions) and Scale 11 (Problems with housing and living conditions). Three further scales met the criterion for all but one question: Scale 4 (Cognitive problems), Scale 7 (Problems with depressed mood) and Scale 10 (Problems with activities of daily living). However, Scale 2 (Non-accidental self-injury) met the criterion for one question only.

The AD index values indicated acceptable and statistically significant agreement between experts, with three exceptions relating to scales that measure behavioural problems – Scale 1 (Overactive or aggressive or disruptive or agitated behaviour), Scale 2 (Non-accidental self-injury) and Scale 3 (Problem drinking or drug-taking).

### Experts’ concerns

Nine themes emerged from experts’ elaborations on their ‘negative’ ratings. These are summarised below, with illustrative quotations.

#### Themes related to comprehensiveness

##### Incomplete coverage

A recurring concern was that the rating descriptors for some scales were not sufficiently specific to older adults:

‘[In] older adults self-harm is often more subtle – not taking medications or accepting required health interventions, isolating or withdrawing from supports’ (Scale 2, Non-accidental self-injury)

‘ … might be worth specifying beyond recommended limits adjusted for age. Perhaps more specifiers for adverse effects, including effects on relationships, self-care, falls’ (Scale 3, Problem drinking or drug-taking)

‘I think this item is too limited in its scope. It does not mention the common types of elder abuse encountered in clinical practice’ (Scale 9, Problems with relationships).

#### Themes related to comprehensibility

##### Lack of fit with clinical thinking

For some scales, experts identified that rating problems separately from the disorders with which they are associated might not fit with usual clinical thinking:

‘Severity of neurocognitive disorder is not just determined by cognitive impairment […] it should include behaviour, self-care, etc.’ (Scale 4, Cognitive problems)

‘ … it would make more sense to include [thought disorder] with other positive psychotic symptoms such as delusions’ (Scale 4, Cognitive problems)

‘Include a sentence to clarify that it is depressed mood not clinical depression that is being rated’ (Scale 7, Problems with depressed mood).

Experts also identified divergence from usual or contemporary conceptualisations for some clinical phenomena:

‘It would be more consistent with clinical reasoning for assessing suicidal risk by adding more risk factors into the descriptors, such as whether having suicidal plans, access to suicidal means, intention to act … ’ (Scale 2, Non-accidental self-injury)

‘There is a move away from “accidental’ versus “intentional” and more towards self-harm in general’ (Scale 2, Non-accidental self-injury).

##### Too many phenomena

Several experts noted that some scales combine too many different phenomena:

‘I have two issues with this item. The first is the conflation of deliberate self-harm with suicidal behaviour … ’ (Scale 2, Non-accidental self-injury)

‘The difficulty is clumping together a range of cognitive problems which may not correspond, e.g. language might be good, memory might be poor. Thought disorder might be prominent, problem solving might be intact’ (Scale 4, Cognitive problems)

with not all included phenomena mentioned in the descriptors for each severity level:

‘Discuss[es] suicide in step 2 but not in step 3 – language needs to be consistent’ (Scale 2, Non-accidental self-injury)

‘Inconsistent exclusion of adverse consequences from rating 3 (included in 2 and 4–5)’ (Scale 4, Cognitive problems).

##### Ambiguity

Some experts indicated ambiguity in the glossary wording:

‘Not clearly identified what the psychological effects of excessive alcohol or substance use may be’ (Scale 3, Problem drinking or drug-taking)

‘Occupation and activities: rating the “quality of meaningful” activities seems rather subjective. This may prove difficult to rate consistently’ (Scale 12, Problems with occupation and activities).

##### Need for more description or examples

Comments about multiple phenomena and ambiguity often corresponded to suggestions for more descriptions or examples to be added to the glossary:

‘It may be useful to expand on what constitutes non-compliant or resistive behaviour’ (Scale 1, Overactive or aggressive or disruptive or agitated behaviour)

‘The scale should have more about IADLs [instrumental activities of daily living] than ADLs [activities of daily living]. In psychiatric care the former are very important – the latter are important but of greater issue for long-term residential care’ (Scale 10, Problems with activities of daily living).

##### Assessment challenges

Assessment challenges were noted for some scales:

‘Sometimes it is difficult to determine what is the most severe problem when there are multiple and almost equally severe problems’ (Scale 8, Other mental and behavioural problems)

‘The problem with the scale is that it requires an independent observation to be rated – that is often not possible, not relevant to the case or occasionally refused’ (Scale 11, Problems with housing and living conditions)

‘Too many judgements here that are likely based on inadequate information’ (Scale 12, Problems with occupation and activities).

#### Themes related to relevance

##### Challenges to capturing change

Some experts expressed concern that some scales lack sensitivity to describe the subtle, delayed or rapid changes often seen in clinical practice:

‘Presentation of a person can change very rapidly, clinical assessment and documentation is more useful in tracking changes of a person's presentation’ (Scale 1, Overactive or aggressive or disruptive or agitated behaviour)

‘Change in dementia is slow and change will not be noticeable within the typical period of clinical contact’ (Scale 4, Cognitive problems).

Others commented on other challenges to capturing change:

‘It could be hard to show change, for example a patient may be elated, with poor sleep and appetite and marked anxiety. Three of the four might improve but the fourth is unchanged – the scale does not alter’ (Scale 8, Other mental and behavioural problems)

‘Some elements of this scale may not be modifiable or changeable if communities have sparse resourcing and groups and transportation is an issue’ (Scale 12, Problems with occupation and activities).

##### Lack of relevance

Some experts considered Scale 12 to be less relevant because of its focus on the environment:

‘In my view, this item is not needed in the scale … Availability of activities is not a patient issue, it's a social system issue’ (Scale 12, Problems with occupation and activities)

or because the instructions about what to include when rating the scale did not cover all relevant treatment contexts:

‘ … would be good to have more mention of residential care situations’ (Scale 11, Problems with housing and living conditions).

#### Need for training

Some comments from experts reinforced the need for training.

‘In New Zealand the cultural context should be emphasized. […] This is important for Māori and Pacific peoples’ (Overarching rating instructions)

‘I find some confusion in the glossary where it states “rate what the person is capable of doing” but then also states “include any lack of motivation”. A person may be capable of doing something but is not doing it because of low motivation’ (Scale 10, Problems with activities of daily living).

### Experts’ summary comments

The survey tasks did not involve comparing the HoNOS OA with the original HoNOS65+. Nonetheless, some experts endorsed the revised title:

‘Well, I notice it's no longer ‘65+’ … I think that's an improvement! I like older adult rather than older persons for example and 65 is stigmatising and misleading … ’.

Others felt the measure had not improved, regardless of revisions:

‘This OA version is not much of an improvement on the 65+ version’.

These mixed views were reflected in comments about the comprehensiveness of the glossary:

‘The content of HoNOS OA includes more detailed descriptions and examples for some of the scales, which are very helpful to rate with confidence’

‘It is too narrow in its focus and some of the items are poorly specified or lacking in range’.

## Discussion

A key finding was that experts held the HoNOS OA scales to be important for determining clinical severity among older adults in contact with specialised mental health services. This accords with studies of the HoNOS65+,^17,18^ and provides reassurance that the glossary revisions have not adversely affected this core aspect of content validity.

Results of the thematic analysis may help explain why ratings of other aspects of content validity were more variable. With respect to comprehensiveness, for example, experts suggested additional older adult-specific examples for some scales – such as not taking medications as a form of self-harm in Scale 2 (Non-accidental self-injury) and elder abuse in Scale 9 (Problems with relationships). This issue may have attracted comment among this sample of experts with a high level of familiarity with the HoNOS65+ glossary, because the wording of some examples was revised to improve consistency between the HoNOS OA and HoNOS 2018.^7^ However, it is important to note that, even in the absence of these older adult-specific examples, the revised glossary provides the opportunity to rate the phenomena of interest (e.g. passive forms of self-harm in Scale 2 and problematic relationships in Scale 9).

With respect to comprehensibility, for example, one concern was that some scales might not reflect usual or contemporary clinical thinking about certain clinical problems. Specifically, some comments suggested it may not be clinically meaningful to rate thought disorder on Scale 4 (Cognitive problems) and depressed mood on Scale 7 (Depressed mood) independently of the disorder(s) with which they are associated. These issues may have attracted comment because the revisions increased the emphasis on rating these phenomena.^7^ For Scale 2 (Non-accidental self-injury), experts commented on how self-injury should be conceptualised. This may reflect an acknowledged lack of consistency in the conceptualisation and description of non-accidental self-injury^19^ and/or difficulties identifying non-accidental self-injury in older adults.^20^

### Implications

Experts rated all HoNOS OA scales as important; this may give clinicians confidence in the measure's relevance to clinical decision-making and care planning. The findings may help inform services in making decisions about implementing the HoNOS OA, noting that other sources of evidence regarding the measure (e.g. interrater reliability, utility and infrastructure costs) are also likely to be needed.

Despite their relative widespread use and generally acceptable measurement properties,^21,22^ some concerns have been raised about the use of the HoNOS as a routine measure of clinical status.^23–25^ There have been calls for further clarification of the construct validity of the HoNOS65+^21,22,26,27^ to guide which scores should be used in practice. Clinicians’ views of the measure's clinical utility have been mixed, with some regarding it as acceptable, but others questioning whether it provides meaningful information to inform clinical practice.^18,21,24,27,28^ Models for using HoNOS65+ scores to inform individual care have been shown to be potentially useful.^29,30^ In our study, experts’ suggestions to expand the older adult-specific examples might raise concerns about the utility of the HoNOS OA. Conversely, including more examples could have adverse effects – encouraging raters to rely on the descriptors as an exhaustive checklist or making the measure unacceptably lengthy. Studies of the measure's utility could explore these possibilities.

Given the breadth of problems covered by the HoNOS OA, training remains critical. Training could helpfully orient experienced clinicians to the rationale for certain revisions, including the reduced emphasis on age-specific examples. Some experts raised concerns about rating some clinical problems independent of disorder. It remains important to emphasise (through training and other means) that the HoNOS OA is not a diagnostic or screening tool.

### Strengths and limitations

This study included experts from two countries with a long history of using the HoNOS65+, lending support for the ‘real-world’ relevance of the results. Survey questions were designed from best practice principles.^9,31^ We used standardised methods to determine whether excellent content validity and acceptable agreement among experts were reached.^12,13,32^ The qualitative component enabled us to explore possible explanations for patterns in the experts’ ratings. However, some limitations should be noted. First, there may have been selection biases. We drew on multiple sources to identify experts and made efforts to confirm their expertise, but did not apply measurable criteria.^33,34^ However, all the experts reported at least one area of HoNOS expertise. Second, there may have been non-response bias as more than a quarter of the invited experts did not complete the survey. We do not know whether those who did not participate held different views from those who did. However, the participating experts expressed a range of views, both positive and negative. Third, to minimise respondent burden, the open-ended questions focused on experts’ concerns. Therefore, any interpretation of the findings should consider the qualitative and quantitative results in tandem.

### Areas for future focus

As well as indicating that the content of the HoNOS OA scales remains important for determining clinical severity among older adults in contact with specialised mental health services, the findings suggest areas for future focus. Training (or other communications) could include a focus on orienting experienced raters to the decreased emphasis on age-specific examples in the glossary; and the findings support progression to evaluating the inter-rater reliability and utility of the HoNOS OA to help address questions about implementation.

## Data Availability

The data-sets generated and analysed for the current study are not available.

## About the authors

**Meredith G. Harris** is a Principal Research Fellow at the School of Public Health at the University of Queensland, Australia, and Researcher, Analysis and Reporting for the Australian Mental Health Outcomes and Classification Network. **Caley Tapp** is a Research Fellow at the School of Public Health at the University of Queensland, Australia, and Researcher, Analysis and Reporting for the Australian Mental Health Outcomes and Classification Network. **Urska Arnautovska** was a Research Fellow at the School of Public Health at the University of Queensland, Australia, and Researcher, Analysis and Reporting for the Australian Mental Health Outcomes and Classification Network at the time the work described in the paper was carried out. **Tim Coombs** is consultant to Training and Service Development, Australian Mental Health Outcomes and Classification Network, Australia. **Rosemary Dickson** is the Network Coordinator for the Australian Mental Health Outcomes and Classification Network, Australia. **Mark Smith** is Programme Lead, Outcomes and Information, at Te Pou, New Zealand. **Angela Jury** is a research manager at Te Pou, New Zealand. **Jennifer Lai** is a researcher at Te Pou, New Zealand. **Mick James** is the national HoNOS advisor at the Royal College of Psychiatrists, London, UK. **Jon Painter** is a senior lecturer in mental health nursing at Sheffield Hallam University, Sheffield, UK. **Philip M. Burgess** is a professor in the School of Public Health at the University of Queensland, Australia, and Lead, Analysis and Reporting for the Australian Mental Health Outcomes and Classification Network.
